# The Unusual Coexistence of Chronic Lymphocytic Leukemia, Dengue Fever, and Acute Kidney Injury: A Case Report

**DOI:** 10.1002/ccr3.73146

**Published:** 2026-08-06

**Authors:** Musawer Khan, Safia Bibi, Zarafshan Khan, F. N. U. Ruqayya, Arwaa Chaudhry, Nauman Khan, Alamzaib Khan, Kamil Ahmad Kamil, F. N. U. Zainullah

**Affiliations:** ^1^ Combined Military Hospital Quetta Quetta Pakistan; ^2^ Quetta Institute of Medical Sciences Quetta Pakistan; ^3^ Federal Medical College Islamabad Pakistan; ^4^ Internal Medicine Department Nasrat Bashir Curative Hospital Kandahar Afghanistan

**Keywords:** AKI, CLL, dengue, peripheral smear, splenomegaly

## Abstract

A 70‐year‐old male with CLL presented with dengue fever complicated by AKI. A diagnostic challenge arose from overlapping features. Investigations showed leukocytosis, lymphocytosis, anemia, thrombocytopenia, positive Anti‐Dengue IgM, and renal impairment. Managed with supportive care, ibrutinib, and monitoring, with improvement. At the 3‐month follow‐up, he remained stable, highlighting the importance of multidisciplinary management.

## Key Clinical Message

The rare coexistence of chronic lymphocytic leukemia, dengue fever, and acute kidney injury underscores the importance of early diagnostic evaluation and multidisciplinary management in immunocompromised patients living in dengue‐endemic regions. Close monitoring and individualized supportive care are essential for optimizing outcomes.

## 1 Introduction

Chronic Lymphocytic Leukemia (CLL) is recognized as the most prevalent form of leukemia in adults, with recent updates to the Surveillance, Epidemiology, and End Results (SEER) database underscoring its status as one of the most common hematologic malignancy [1]. This condition arises from spontaneously acquired mutations leading to the accumulation of abnormal B‐lymphocytes in the blood, bone marrow, lymph nodes, and spleen, manifesting in hallmark symptoms such as lymphadenopathy, splenomegaly, and cytopenias [2]. Diagnosis is typically established through differential blood counts, peripheral blood smears, and, in many cases, bone marrow biopsy to assess the extent of marrow involvement [3]. In contrast, Dengue Fever, caused by the Dengue virus, a member of the Flaviviridae family transmitted by the *Aedes aegypti* mosquito, presents with a spectrum of symptoms including high fever, severe headache, retro‐orbital pain, joint pain, and rash, which may progress to severe forms such as Dengue Hemorrhagic Fever (DHF) or Dengue Shock Syndrome (DSS) [4, 5]. While CLL and Dengue Fever are infrequently reported together, their coexistence introduces significant clinical complexity due to overlapping symptoms and distinct pathophysiological mechanisms, including immune dysregulation and systemic inflammation. The addition of Acute Kidney Injury (AKI) in this context further complicates management, potentially stemming from hypovolemia, renal vasculitis, or the effects of systemic inflammation associated with both conditions [6, 7]. This case report describes a patient diagnosed with CLL who developed Dengue Fever complicated by AKI, shedding light on the diagnostic challenges, therapeutic strategies, and the rare confluence of these three conditions in a resource‐limited, Dengue‐endemic setting.

### 1.1 Patient Information

A 70‐year‐old male shopkeeper from Quetta, Pakistan, presented to the emergency department on January 12, 2025, with a 1‐month history of weight loss, generalized weakness, and loss of appetite, followed by high‐grade fever, rigors, chills, myalgia, nausea, vomiting, and oliguria for 3 days. He had no prior medical or surgical history, was a nonsmoker, and reported no alcohol use. Vaccination history was unremarkable. The patient had recently traveled to Khairpur, Sindh, and reported significant mosquito exposure prior to the onset of symptoms. Family history was unremarkable for hypertension, diabetes, or hereditary diseases.

### 1.2 Clinical Findings

**FIGURE 1:**
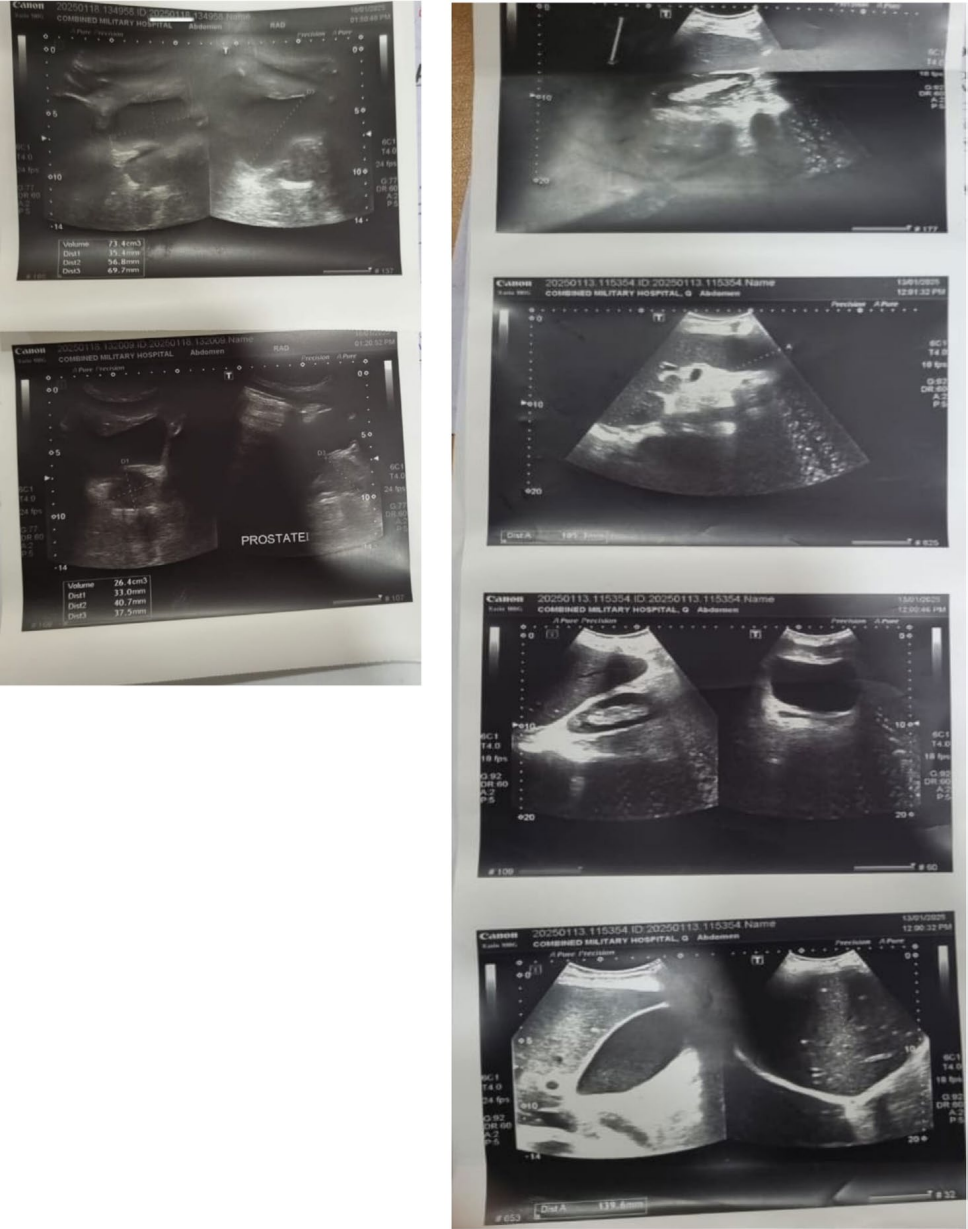
Ultrasound (USG) of the abdomen and prostate (pre and post void urine). Transverse view showing splenomegaly (spleen measuring 18.5 cm, arrow indicating spleen border) with normal‐sized kidneys exhibiting increased parenchymal echogenicity. Mildly enlarged prostate (26 cm^3^) with post‐void residual volume of 70 mL is noted. No ascites or focal lesions observed. (Image courtesy of Combined Military Hospital Quetta; patient identifiers removed).

On admission, vital signs were: blood pressure 118/65 mmHg, pulse 96 bpm, respiratory rate 18 breaths/min, temperature 101°F, SpO_2_ 95% on room air, and random blood sugar 142 mg/dL. The patient appeared ill but was conscious and oriented. Examination revealed cervical and inguinal lymphadenopathy, right‐sided shoulder and hip pain with restricted range of motion, and a palpable spleen 3 cm below the costal margin (confirmed by ultrasound; Figure 1). Systemic examination (cardiovascular, respiratory, central nervous, and gastrointestinal) was unremarkable. The clinical course and interventions are summarized in Table 1.

**TABLE 1:** Timeline of clinical presentation and interventions.

Sr.#	Date	Event	Event
1.	December 2024	Symptom onset	Weight loss, fatigue, loss of appetite noted
2.	January 12, 2025	Hospital admission	Admitted for fever, myalgia, nausea, vomiting, and oliguria
3.	January 12, 2025	Initial treatment	Administration of IV fluids, antipyretics, antiemetics; Dengue IgM and blood work ordered
4.	January 15, 2025	Bone marrow biopsy	Performed to confirm CLL diagnosis
5.	January 16, 2025	Referral	Referred to hematology and nephrology for multidisciplinary management
6.	April 2025	Follow‐up	Outpatient review showed improved symptoms and renal function

### 1.3 Diagnostic Assessment

**FIGURE 2:**
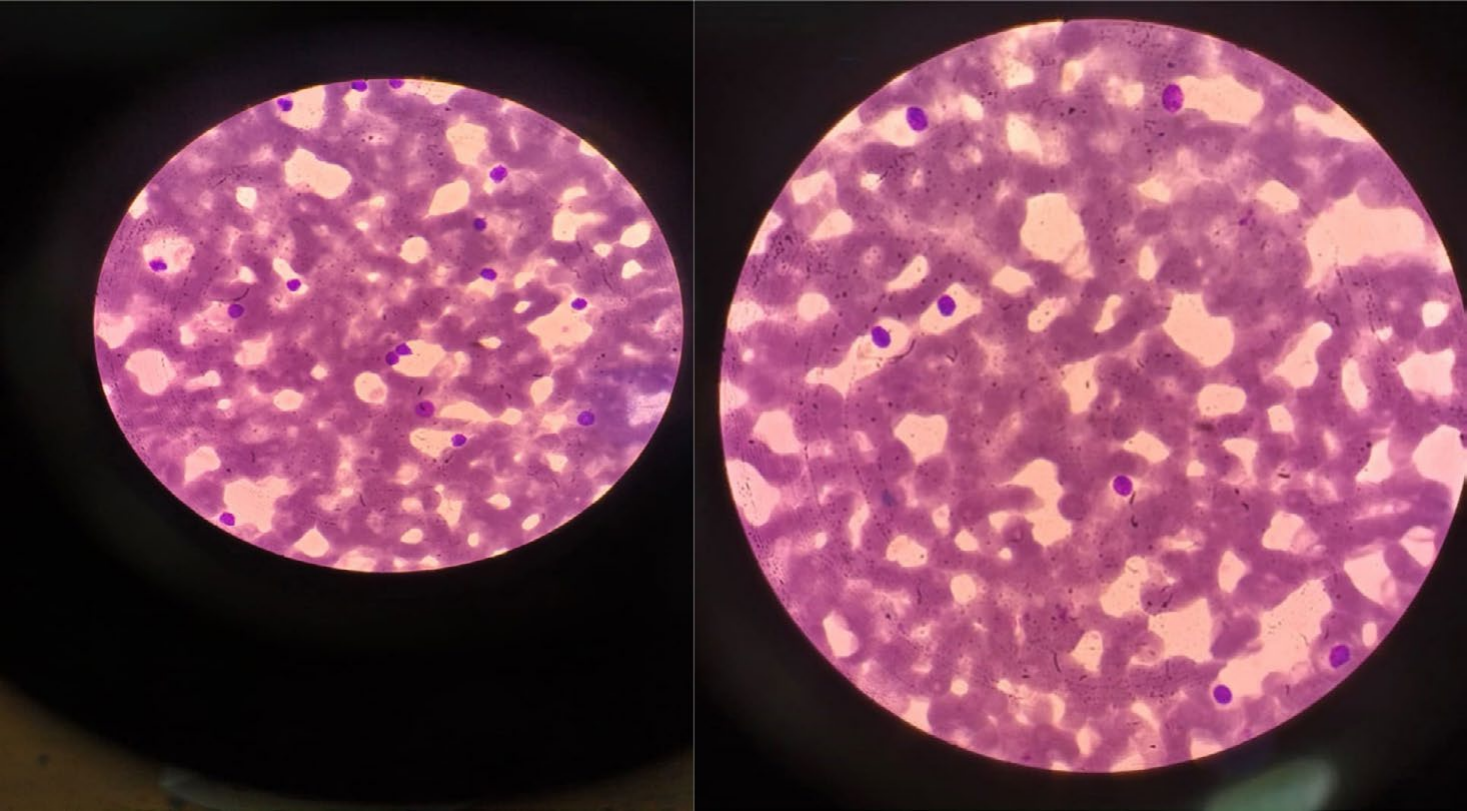
Peripheral blood smear slide. Wright‐Giemsa stain demonstrating a dense population of small, mature lymphocytes with smudge cells, consistent with chronic lymphocytic leukemia (CLL). (Image courtesy of Combined Military Hospital Quetta; patient identifiers removed).

Laboratory results showed leukocytosis (total leukocyte count 285.7 × 10^9^/L, 98% lymphocytes), anemia (hemoglobin 7.9 g/dL, hematocrit 0.27 L/L), and thrombocytopenia (platelets 80 × 10^9^/L). Peripheral smear revealed polychromasia, macrocytosis, anisocytosis, marked lymphocytosis, and smudge cells, diagnostic of CLL (Figure 2). Bone marrow biopsy confirmed hypercellular marrow with 68% lymphocytes, megaloblastic erythropoiesis, and decreased iron stores. Anti‐Dengue IgM was positive, confirming Dengue Fever. AKI was evident (serum creatinine 253 μmol/L, urea 21 mmol/L, eGFR 23 mL/min/1.73 m^2^), with urinalysis showing proteinuria and hematuria (8–14 RBCs/HPF). Abdominal ultrasound demonstrated splenomegaly (18.5 cm) with normal‐sized kidneys and increased parenchymal echogenicity (Figure 1). High‐resolution CT confirmed bilateral inguinal and small mediastinal lymphadenopathy. Elevated C‐reactive protein (265 mg/L) and lactate dehydrogenase (256 U/L) supported systemic inflammation.

### 1.4 Therapeutic Intervention

Supportive care for Dengue included IV crystalloids titrated to hemodynamic status and urine output, with antipyretics and antiemetics. Platelet transfusions were given for thrombocytopenia (< 80 × 10^9^/L) to reduce bleeding risk. Ibrutinib 420 mg daily was started for symptomatic CLL, per hematology guidance. AKI management involved careful hydration and monitoring, with nephrology consultation to avoid nephrotoxic agents (e.g., contrast, aminoglycosides). Hemodialysis was not required as creatinine stabilized.

### 1.5 Follow‐Up and Outcomes

At three‐month follow‐up (April 2025), the patient reported resolution of fever, myalgia, and nausea, with improved appetite and energy. Labs showed stable hemoglobin (8.5 g/dL), reduced lymphocyte count (50 × 10^9^/L), and improved renal function (creatinine 120 μmol/L, eGFR 45 mL/min/1.73 m^2^). Ultrasound confirmed reduced splenomegaly (14 cm). The patient adhered to ibrutinib and follow‐up, with no significant adverse events.

## 2 Discussion

Dengue virus, a member of the Flaviviridae family, poses a significant threat in endemic regions, particularly among immunocompromised individuals such as patients with hematological malignancies [8]. Despite its potential severity, evidence regarding dengue infection in this population remains limited [9].

This case suggests that dengue infection in patients with hematological diseases may exhibit atypical clinical manifestations and disease progression compared with the general population [9], largely due to underlying immunosuppression. In this case, chronic lymphocytic leukemia (CL) associated immune dysfunction likely increased susceptibility to infection, while dengue fever subsequently contributed to the development of acute kidney injury (AKI) through multifactorial mechanisms. To our knowledge, the coexistence of CLL, dengue fever, and AKI has rarely been reported in the literature, with only limited documentation in the literature, making this case a valuable contribution to the understanding of atypical presentations in immunocompromised patients residing in dengue‐endemic regions.

CLL is associated with an increased risk of renal complications through several potential mechanisms, including leukemic infiltration, immune‐mediated glomerular disease, and treatment‐related nephrotoxicity [10, 11, 12]. Furthermore, the immunosuppressive state associated with CLL predisposes patients to infections, including viral illnesses. Previous studies suggest that dengue fever in immunocompromised individuals, such as those with CLL, may deviate from the typical biphasic course and instead may present with prolonged viral clearance, atypical organ involvement, and severe complications such as AKI [6, 7]. In the present case, AKI likely resulted from multiple contributing factors, including dengue‐induced hypovolemia and capillary leakage, compounded by underlying renal vulnerability related to CLL, such as possible interstitial leukemic infiltration or therapy‐associated nephrotoxicity [5, 13, 14, 15]. The peripheral blood smear (Figure 2) and dengue NS1 antigen testing during early febrile phase were instrumental in establishing a timely diagnosis, consistent with recommendations supporting rapid diagnostic strategies in endemic settings [7, 14]. IgM Testing and Flow Cytometry could not be performed due to resource limitations at our institution.

Management required a multidisciplinary approach involving hematology for CLL management with ibrutinib, infectious disease support for dengue management, and nephrology oversight for AKI, including careful fluid resuscitation [16] and avoidance of nephrotoxic agents [7, 14]. This collaborative strategy was associated with clinical and laboratory improvement at follow‐up. Existing literature, including reports involving transplant recipients with dengue infection, similarly suggests that immunocompromised hosts may experience more severe disease courses, emphasizing the need for heightened clinical vigilance [13].

This case has several limitations. The follow‐up period was limited to 3 months, restricting long‐term outcome assessment. Additionally, molecular profiling, such as IGHV mutation status, was unavailable and may have provided further characterization of the CLL subtype. The absence of renal biopsy also limited definitive determination of the precise mechanism underlying AKI. Future studies involving larger cohorts are needed to better elucidate the interaction between dengue infection, hematological malignancies, and renal complications, potentially guiding preventive strategies and individualized management approaches. Overall, this case highlights the importance of early diagnostic preparedness and interdisciplinary care in managing rare and complex clinical presentations in dengue‐endemic regions.

### 2.1 Differential Diagnoses

At presentation, several differential diagnoses were considered. Given the endemic setting, malaria and typhoid fever were initially suspected as common causes of febrile illness with cytopenias. However, peripheral smear and blood cultures excluded these. Acute viral infections such as chikungunya and Epstein–Barr virus were also considered, but the positive anti‐dengue IgM and classical clinical features supported Dengue Fever. Hematologic differentials included acute lymphoblastic leukemia and other lymphoproliferative disorders; however, the characteristic peripheral smear with smudge cells and bone marrow findings confirmed Chronic Lymphocytic Leukemia (CLL). The acute kidney injury was initially attributed to pre‐renal causes such as hypovolemia, but the possibility of intrinsic causes including leukemic infiltration and dengue‐associated nephropathy was carefully evaluated by nephrology.

Given the patient's fever, systemic inflammation, and AKI, sepsis and hemophagocytic lymphohistiocytosis (HLH) were also considered. However, the absence of a definite infectious focus and features suggestive of bacterial sepsis, together with a positive dengue NS1 antigen and characteristic hematological findings, supported dengue fever as the primary diagnosis. HLH was considered less likely because there was no evidence of hemophagocytosis on bone marrow examination or other characteristic clinicopathological features.

### 2.2 Patient Perspective

The patient stated, “I initially thought I had a simple fever, so learning that I had Chronic Lymphocytic Leukemia was unexpected and made me feel anxious. The fever and weakness were overwhelming, but the healthcare team explained my condition clearly and started treatment promptly, which reassured me. My treatment and referral were provided through government healthcare services, so I did not experience a financial burden. As I recover, I now feel much better and more confident about managing my health.”

## 3 Conclusion

A rare sequence of chronic lymphocytic leukemia, dengue fever, and acute kidney injury highlights the importance of early diagnostic testing and multidisciplinary care in immunocompromised patients in dengue‐endemic regions. Vigilant monitoring and tailored supportive management are crucial to improve outcomes.

Immunocompromised patients with chronic lymphocytic leukemia may present with atypical or severe dengue infection complicated by acute kidney injury. In dengue‐endemic regions, persistent lymphocytosis, splenomegaly, or unexplained cytopenias should prompt evaluation for underlying hematologic malignancy. Early multidisciplinary management and careful renal monitoring are essential for favorable outcomes.

## Author Contributions

**Alamzaib Khan:** resources, investigation, supervision, methodology. **Arwaa Chaudhry:** writing – original draft, writing – review and editing, validation, visualization. **F. N. U. Zainullah:** methodology, visualization, investigation, writing – review and editing. **Safia Bibi:** supervision, validation, resources. **Nauman Khan:** methodology, writing – original draft, validation. **Kamil Ahmad Kamil:** visualization, writing – original draft, validation. **F. N. U. Ruqayya:** writing – original draft. **Zarafshan Khan:** resources, investigation, supervision, writing – original draft. **Musawer Khan:** conceptualization, data curation, supervision, project administration.

## Funding

The authors have nothing to report.

## Ethics Statement

Ethical approval was not required for this case report in accordance with the policies of our institution and local regulations.

## Consent

Written informed consent was obtained from the patient for publication of this case report and the accompanying images.

## Conflicts of Interest

The authors declare no conflicts of interest.

## Data Availability

The data supporting the findings of this study are available within the manuscript.
